# Secreted gelsolin desensitizes and induces apoptosis of infiltrated lymphocytes in prostate cancer

**DOI:** 10.18632/oncotarget.20414

**Published:** 2017-08-23

**Authors:** Chun-Chi Chen, Shiow-Her Chiou, Cheng-Lin Yang, Kuan-Chih Chow, Tze-Yi Lin, Hui-Wen Chang, Weir-Chiang You, Hisu-Wen Huang, Chien-Min Chen, Nien-Cheng Chen, Fen-Pi Chou, Ming-Chih Chou

**Affiliations:** ^1^ Institute of Medicine, Chung-Shan Medical University, Taichung, Taiwan; ^2^ Section of Urology, Departments of Surgery, Changhua Christian Hospital, Chang-Hua, Taiwan; ^3^ Graduate Institute of Microbiology and Public Health, National Chung Hsing University, Taichung, Taiwan; ^4^ Graduate Institute of Biomedical Sciences, National Chung Hsing University, Taichung, Taiwan; ^5^ Department of Pathology, China Medical University Hospital, China Medical University, Taichung, Taiwan; ^6^ Department of Radiation Oncology, Taichung Veterans General Hospital, Taichung, Taiwan; ^7^ Endemic Species Research Institute, Council of Agriculture, Executive Yuan, Chi-Chi, Taiwan; ^8^ Institute of Biochemistry, Microbiology and Immunology, Chung-Shan Medical University, Taichung, Taiwan; ^9^ Department of Family and Community Medicine, Chung-Shan Medical University Hospital, Chung-Shan Medical University, Taichung, Taiwan

**Keywords:** prostate cancer, gelsolin, sortilin, CD37, tumor-infiltrated lymphocytes

## Abstract

Loss of immunosurveillance is a major cause of cancer progression. Here, we demonstrate that gelsolin, a constituent of ejaculate, induces apoptosis of activated lymphocytes in prostate cancer. Gelsolin was highly expressed in prostate cancer cells, and was associated with tumor progression, recurrence, metastasis, and poor prognosis. *In vitro*, secreted gelsolin inactivated CD4^+^ T cells by binding to CD37, and induced apoptosis of activated CD8^+^ T lymphocytes by binding to Fas ligand during cell contact dependent on major histocompatibility complex I. Moreover, secreted gelsolin bound to sortilin, which in turn bound to Wiskott-Aldrich syndrome protein family member 3, thereby enhancing the endocytosis and intracellular transport of essential lipids needed to facilitate tumor growth and expansion. Under normal conditions, gelsolin is a seemingly harmless protein that prevents immune responses in female recipients. In disease states, however, this protein can inhibit immunosurveillance and promote cancer progression.

## INTRODUCTION

Prostate cancer (PCa) is the second-leading cause of cancer-related death in the USA male patients, which accounts for about 10% of cancer-related deaths [1]. The annual death rate of PCa is 24.7 per 100,000 men. In Scandinavian and Baltic countries, although the incidence rate of PCa is equivalent to that in central Europe and USA, the mortality rate is much higher, average around 34.5/10^5^ men [1]. In Taiwan, although the annual mortality rate of PCa (8.6/10^5^ men in 2015) is lower than that in the Euro-American area, the incidence rate and the mortality rate have respectively increased about 3- and 4-fold in the past two decades (Annual reports of the Ministry of Health and Services, Taiwan, 2012).

For patients with early-stage PCA, the treatment results of prostatectomy and androgen deprivation are generally satisfactory. For patients with locally advanced cancers, palliative radiation therapy can often achieve appropriate disease control. However, in patients with more advanced diseases, particularly those with bone metastasis (~70% of advanced PCa patients) [2, 3], even androgen-sensitive tumor cells become resistant to radiation and conventional chemotherapeutic agents (e.g., Adriamycin, cisplatin, etoposide, 5-fluorouracil, methotrexate, mitoxantrone, vincristine and vinblastine) [4]. However, the mechanisms underlying such spontaneous resistance to radiation and anticancer drugs, as well as the propensity for bone metastasis, are not well understood.

Our previous studies demonstrated that AKR1C2, an isoform of aldo-keto reductase 1C (AKR1C), was frequently detected in PCa patients and was associated with disease status, tumor grade and androgen receptor expression [5]. *In vitro*, AKR1C2 expression correlated with resistance to anticancer drugs and lycopene. Interestingly, AKR1C2 induced the expression of hepatocyte growth factor and interleukin 8 when cells were exposed to hypoxic conditions [6]. Hepatocyte growth factor, in turn, upregulated ATPase AAA domain containing 3A (ATAD3A), a prospective intracellular-transport-related ATPase and an anti-apoptotic factor [7] associated with the import of nuclear DNA repair-related enzymes and the release of prostate-specific antigen (PSA) [8]. PSA (kallikrein-3) is a kallikrein-related peptidase that is normally present in the ejaculate to liquefy semen in the seminal coagulum and dissolve cervical mucus, allowing sperm to swim freely and enter the uterus copiously [9]. In patients with prostatitis, benign prostatic hyperplasia or PCa, serum PSA levels are elevated because of the local obstructions and the limited accessibility to the secretory ducts [10].

Besides PSA, another secreted protein, gelsolin, is also associated with prostate tumorigenesis and malignant transformation [11]. Two forms of gelsolin protein, cytosolic gelsolin and secreted gelsolin, are transcribed from a single gene [12]. Cytosolic gelsolin collaborates with villin in the capping and severing of actin and the formation of F-actin (depolymerization and polymerization of actin filaments during cell movement), and has been the focus of most studies [11, 13]. The role of secreted gelsolin in PCa, however, has not been examined.

Here we examined the expression of gelsolin in PCa specimens and its effects on PCa progression. *In vitro*, we explored the impact of increased gelsolin expression on responsiveness to radiation and anticancer drug treatment. We also studied the effects of secreted gelsolin on infiltrating T cell sensitivity, and on cholesterol uptake *via* sortilin, an oncogene-associated receptor on PCa cells [14].

## RESULTS

### Gelsolin expression correlates with disease progression and clinical outcomes in PCa patients

**Figure 1:**
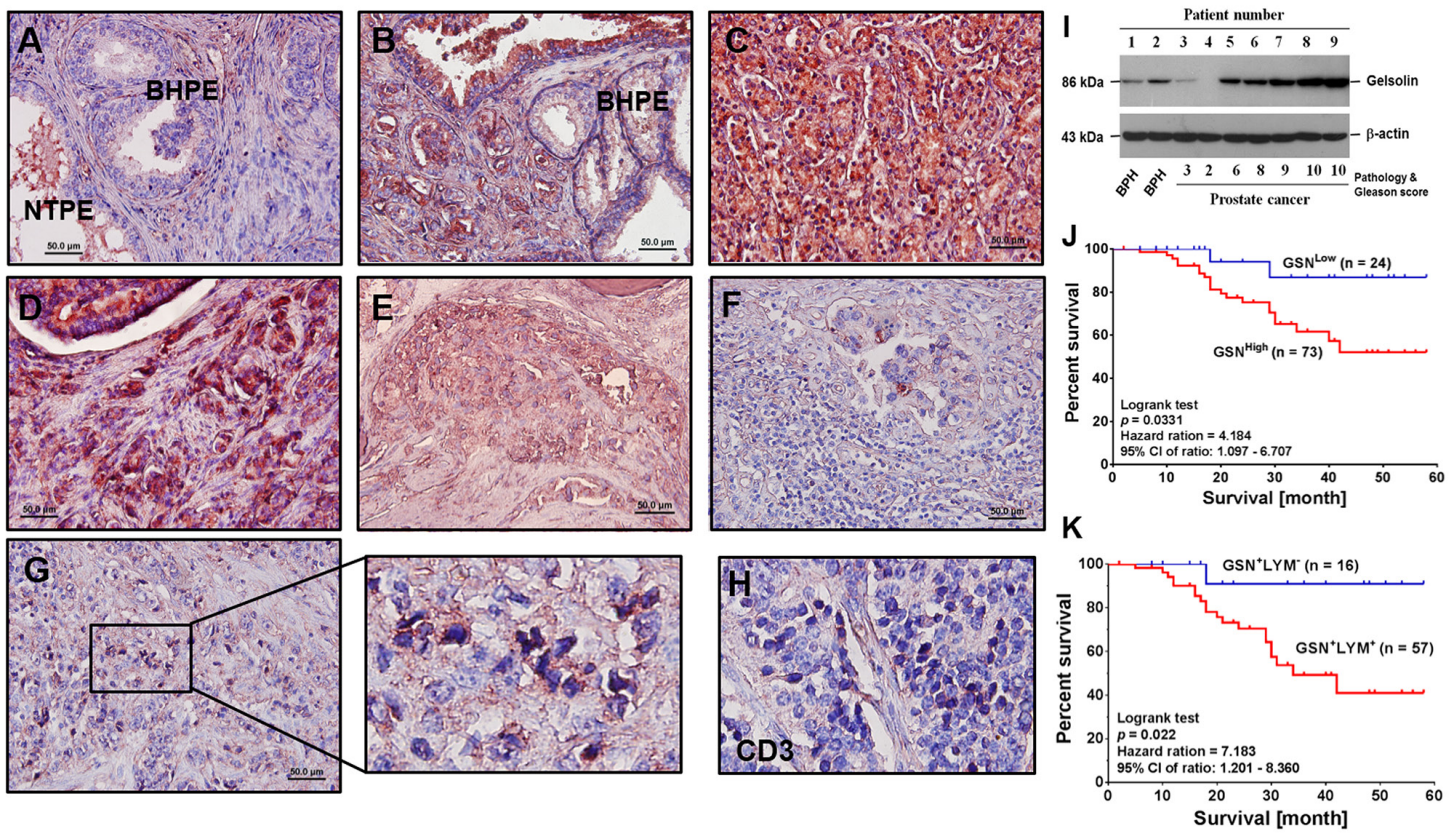
Expression of gelsolin is associated with poor clinical outcomes in patients with PCa **(A)** Representative examples of gelsolin (red crimson precipitates) expression in prostate cancer (PCa) cells, which was detected by immunohistochemistry, and the slide was counterstained with hematoxylin. In non-tumor prostate epithelial cells, secreted gelsolin was detected in the lumens (original magnification × 100). **(B)** Compared to hypertrophic region, expression of gelsolin was detected in tumor nests (Gleason score 6) (original magnification × 100). **(C)** In pathological tissue of PCa specimens with higher Gleason score, overexpression of gelsolin was detected in all the PCa cells (Gleason score 9) (original magnification × 100). **(D)** In PCa tissue with metastatic lesions, overexpression of gelsolin was detected in tumor nest and the metastatic PCa cells (Gleason score 9) (original magnification × 100). **(E)** In bone marrow section of PCa specimens, gelsolin was mainly detected in the invasive PCa cells (original magnification × 100). **(F)** Around tumor nest, in which gelsolin was minimally expressed, a swarm of lymphocytes without evident gelsolin signal were surrounding the tumor nest (original magnification × 100). **(G)** In some areas, many cells with darkly stained nuclei in the tumor nest were positive for gelsolin. However, nuclear morphologies of these cells were different from the neighboring PCa cells (original magnification × 400). **(H)** Similar to cells, which had darkly stained nuclei in PCa tumor nest, the cells were CD3-positive (original magnification × 400). **(I)** Expression of gelsolin in PCa and benign hypertrophic prostate epithelia (BHPE) cells was confirmed by Western blotting analysis. **(J)** Kaplan-Meier plots of overall survival in PCa patients with high (GSN^High^) or low gelsolin (GSN^Low^) expression. **(K)** Survival analysis of PCa patients in whom lymphocytes were positive (GSN^+^LYM^+^) or negative (GSN^-^LYM^-^) for gelsolin staining. Detection of gelsolin staining correlated with poor prognosis.

To determine the function of gelsolin in PCa, we recruited a cohort of 97 patients and measured gelsolin expression in pathological sections by immunohistochemical staining. Compared to non-tumorous prostate epithelia (Figure 1A) and benign hypertrophic prostate epithelia (Figure 1A and 1B), PCa tissues (Figure 1B-1D) and metastatic bone marrow (Figure 1E) highly expressed gelsolin. In some PCa tissues, in which gelsolin was not highly expressed, clear swarms of tumor-infiltrated lymphocytes (TIL) were detected around the tumor nests (Figure 1F). However, within some PCa tissues, the gelsolin -positive cells (Figure 1G) had darker nuclei (amplified part of Figure 1G) and were CD3-positive (Figure 1H), indicating that these cells were T lymphocytes. Interestingly, the nuclei were smaller, darkly stained and spiky, suggesting that these cells could be apoptotic.

Gelsolin expression in PCa cells was confirmed by Western blotting analysis (Figure 1I). Statistical results showed that gelsolin expression in PCa was associated with disease status, tumor grade, cigarette smoking, serum PSA level, lymphovascular infiltration and the expression of androgen receptor, AKR1C2 and epidermal growth factor receptor (EGFR) (Table 1). Thus, gelsolin expression correlated with the growth and invasive potential of PCa cells.

**Table 1:** Association of gelsolin expression with clinicopathologic parameters in patients with prostate cancer in Taiwan

Clinicopathologic parameters	Expression of gelsolin		
**(n, number of patients)**	**Low (n = 24)**	**High (n = 73)**	***p* value**
Age (48-83 years)	68.4 ± 5.5	66.9 ± 6.1	0.742*
Disease status			
Localized (stage A or B) (n = 57)	11	46	0.0118^‡^
Advanced (Stage C or D) (n = 29)	6	23	
Undetermined (n = 11)	7	4	
Tumor grade			
Gleason < 7 or well or moderately differentiated (n = 65)	13	52	0.0182^‡^
Gleason ≥ 7 or poorly differentiated (n = 27)	7	20	
Undetermined (n = 5)	4	1	
Cigarette Smoking			
Smoker (n = 69)	7	62	< 0.001^‡^
Non-smoker (n = 28)	17	11	
Lymphovascular infiltration			
Positive (n = 29)	1	28	0.0015^‡^
Negative (n = 68)	23	45	
Serum PSA (ng/ml)			
≤ 10 (n = 21)	9	12	0.0298^‡^
>10 (n = 76)	15	61	
Expression of androgen receptor			
High (n = 81)	15	66	0.0014^†^
Low (n = 16)	9	7	
Expression of ATAD3A			
High (n = 82)	11	71	< 0.001^‡^
Low (n = 15)	13	2	
Expression of aldo-keto reductase 1C2			
High (n = 86)	17	69	0.0043^‡^
Low (n = 11)	7	4	
Expression of EGFR			
High (n = 79)	13	66	< 0.001^‡^
Low (n = 18)	11	7	
Expression of cyclooxygenase-2			
High (n = 27)	10	17	0.0814^‡^
Low (n = 70)	14	56	

A smoker was defined as a person who smoked more than 20 packs (> 1 cigarette/day) per year.

*Two-sided *p* value determined by Mann-Whitney test

^†^Two-sided *p* value determined by χ^2^ test for trend

^‡^Two-sided *p* value determined by χ^2^ test (Fisher's exact test was used when the number in one of the cells was equal to or smaller than 5).

In survival analysis, gelsolin overexpression in PCa patients was associated with poor prognosis (Figure 1J), particularly in patients whose lymphocytes were positive for gelsolin (Figure 1K). When gelsolin expression was compared immunohistochemically between Taiwanese and American PCa specimens, gelsolin was detected in 73 (75.25%) of 97 Taiwanese sections and 136 (74.31%) of 183 American samples. There was no evident difference between the samples of these two cohorts (*p* = 0.863; odds ratio = 0.951; relative risk, 0.963). Gelsolin expression also correlated with disease status and tumor grade of American PCa patients as well (Table 2).

**Table 2:** Association of gelsolin expression with clinicopathologic parameters in patients with prostate cancer in the USA

Clinicopathologic parameters	Expression of gelsolin		*p* value
(number of patients)	Low (n = 47)	High (n = 136)	
Disease status			
Localized (stage A or B) (n = 82)	18	64	0.0159^‡^
Advanced (Stage C or D) (n = 74)	15	59	
Undetermined (n = 27)	14	13	
Tumor grade			
Gleason < 7 or well or moderately differentiated (n = 69)	26	43	0.038^†^
Gleason ≥ 7 or poorly differentiated (n = 114)	21	93	

^†^Two-sided *p* value determined by χ^2^ test for trend

^‡^Two-sided *p* value determined by χ^2^ test (Fisher's exact test was used when the number in one of the cells was equal to or smaller than 5).

### The majority of gelsolin is secreted *via* the intracellular export system

**Figure 2:**
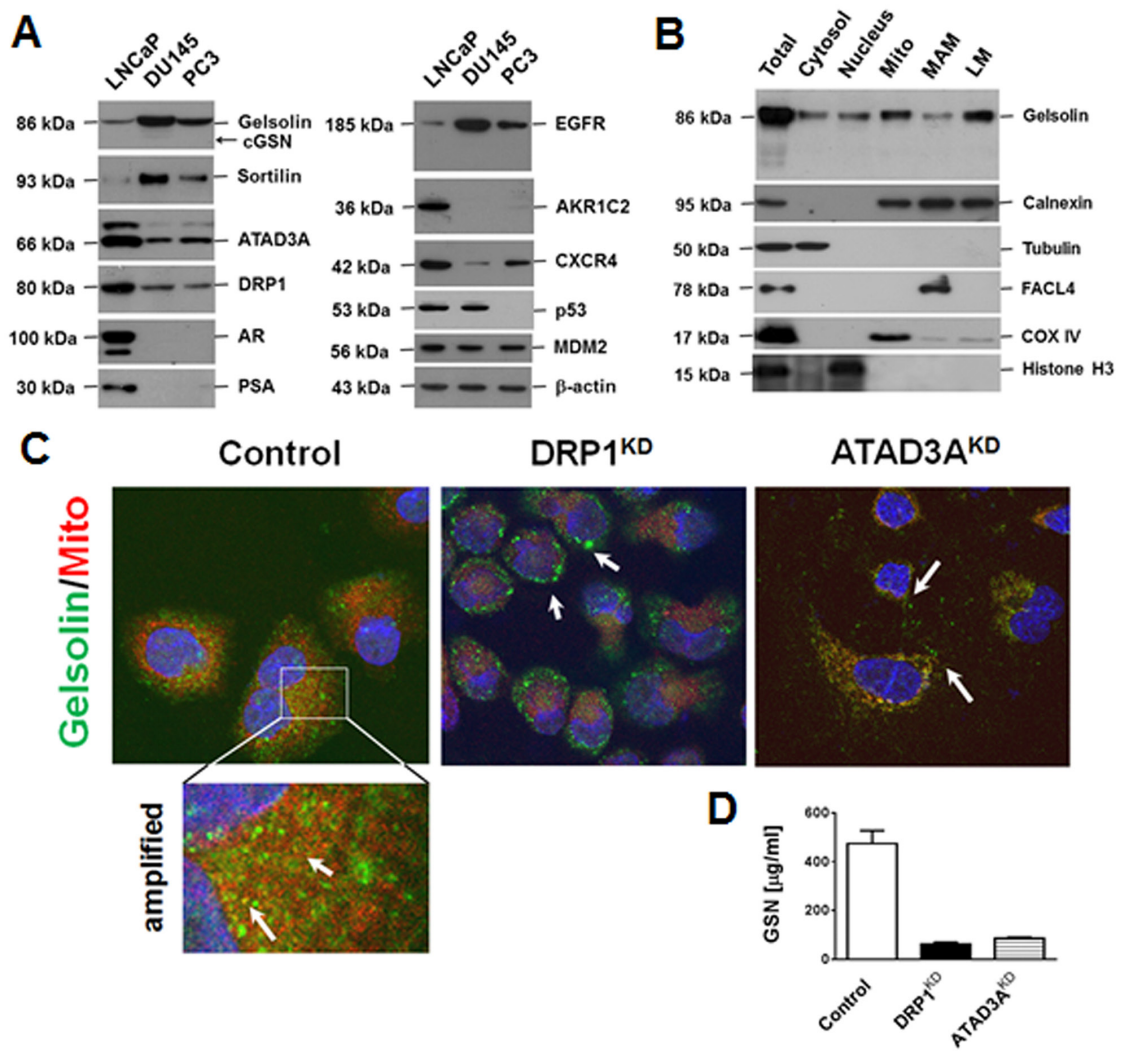
Gelsolin expression in PCa cell lines, and the intracellular locations and extracellular levels of gelsolin **(A)** Expression of gelsolin and tumor-associated proteins in three PCa cell lines, LNCaP, DU145 and PC3, were determined by Western blotting. Gelsolin expression correlated positively with sortilin and EGFR expression, but negatively with ATAD3A and DRP1 expression (essential proteins for intracellular transport [7]). **(B)** Percoll self-generating gradient fractionation was used to localize gelsolin in the fractions of cytosol, nucleus, mitochondria-associated membrane (MAM), mitochondria, and light membranes (LM, majority is microsomes), suggesting that most of gelsolin was present in membrane structures. **(C)** Confocal immunofluorescence micrographs revealed that gelsolin was localized in vesicles, and some of gelsolin signal overlapped with the mitochondrial marker (Control, the left panel and the amplified region). Nuclei were stained with 4’,6-diamidino-2-phenylindole (DAPI). Knockdown of DRP1 (DRP1^KD^) increased the numbers of vacuoles, suggesting that the ER or MAM were enlarged (center panel). When ATAD3A was knocked down (ATAD3A^KD^), mitochondrial staining was reduced and the gelsolin signals were randomly distributed in the cytoplasmic vesicles (right panel). **(D)** Silencing of ATAD3A or DRP1 markedly reduced extracellular gelsolin levels in culture media of LNCaP cells. The results are shown as the means ±standard deviations of three independent experiments. Monoclonal antibodies to DRP-1 and ATAD3A were self-grown.

Three PCa cell lines, PC3, DU145 and LNCaP, were examined by western blotting for the expression of gelsolin and the other tumor-associated proteins. As shown in Figure 2A, gelsolin, EGFR, mouse double minute 2 homolog (MDM2) and the enzymes of intracellular transport system (e.g., ATAD3A and dynamin-related protein 1 [DRP-1] were detected in all three PCa cell lines. Levels of 86-kDa gelsolin were much higher in DU145 (15-fold) and PC3 (6-fold) cells than in LNCaP cells. Interestingly, gelsolin levels were inversely proportional to ATAD3A and DRP-1 levels. The alternately spliced 68-kDa cytosolic gelsolin was only detected in DU145 cells, and its expression was about 1000 times lower than that of 86-kDa gelsolin. An enzyme-linked immunosorbent assay revealed that the levels of secreted gelsolin in culture media from LNCaP cells (474.7 ± 30.6 μg/ml) were significantly higher than those from DU145 (1.30 ± 0.26 μg/ml) or PC3 (21 ± 4 μg/ml) cells, supporting the pathological observations of some of gelsolin in the extracellular space. Furthermore, the expression of gelsolin was proportional to that of sortilin (also known as neurotensin receptor-3, NTS3) and EGFR (Figure 2A).

Western blotting analysis of sucrose gradient-separated organelle fractions (Figure 2B) and a MitoTracker® Red CMXRos uptake assay with confocal fluorescence immunocytochemistry (Figure 2C) clearly demonstrated that gelsolin was mostly localized in light membrane (LM) and mitochondria-associated membrane (MAM) fractions of the endoplasmic reticulum (ER) besides the mitochondrial (Mito) fraction (yellow fluorescence in the amplified portion of Figure 2C). Knockdown of DRP1 (DRP1^KD^) increased a number of enlarged vacuole-like structures, which were strongly positive for gelsolin (center square, Figure 2C) [15]. Knockdown of ATAD3A expression (ATAD3A^KD^), in contrast, reduced the overlapping signals from gelsolin (green fluorescence) and mitochondria (red fluorescence), but increased the numbers of small vesicles [7], some of which could be located extracellularly (right square, Figure 2C). In culture media of DRP1^KD^ or ATAD3A^KD^ cells, the level of secreted gelsolin was much lower than that of the control cells (Figure 2D). The findings were consistent with the results of a web program (http://psort.hgc.jp/) predicting that full-length gelsolin (GenBank: AK315494) carries an N-terminal signal sequence, is synthesized in the ER, is transported to the plasma membrane and is secreted into the extracellular space as secreted gelsolin (Supplementary Figure 1).

### Gelsolin expression correlated with cell migration and resistance to anticancer drugs

**Figure 3:**
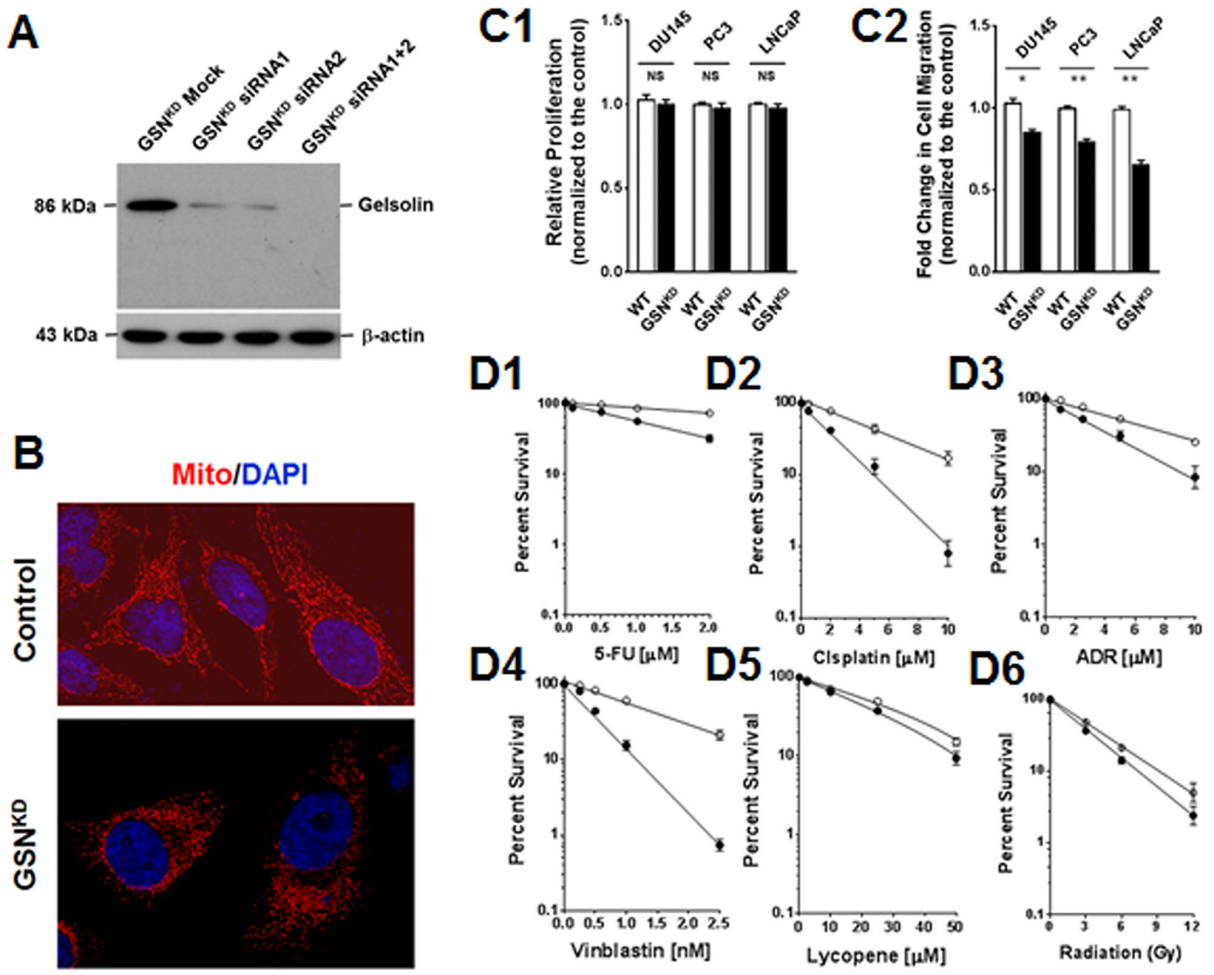
Gelsolin expression correlated with cell migration and resistance to anticancer drugs **(A)** Knockdown of gelsolin expression (GSN^KD^), as shown by Western blotting analysis. **(B)** Increased mitochondrial fragmentation was clearly detected in GSN^KD^ DU145 cells. **(C)** The influence of gelsolin on cell proliferation and mobility. **(C1)** Relative cell growth was only slightly reduced in GSN^KD^ cells. **(C2)** Cell migration, however, was significantly reduced in GSN^KD^ cells. **(D)** Silencing of gelsolin reduced cell resistance to **(D1)** 5-fluorouracil (5-FU), **(D2)** cisplatin, **(D3)** Adriamycin (ADR) and **(D4)** vinblastine (VBL). However, **(D5)** lycopene and **(D6)** radiation had smaller effects on GSN^KD^ DU145 cells.

Knockdown of gelsolin expression (GSN^KD^; Figure 3A) increased mitochondrial fragmentation (Figure 3B), and significantly reduced cell growth (Figure 3C1) and cell migration (Figure 3C2). Resistance to 5-fluorouracil, cisplatin, adriamycin and vinblastine were also reduced in GSN^KD^ cells (Figure 3D1-3D4), corresponding well with previous reports that gelsolin expression was associated with the metastatic potential and anticancer drug resistance of cancer cells [11, 16, 17]. However, gelsolin expression had less of an effect on sensitivity to lycopene and radiation (Figure 3D5 and 3D6).

### Secreted gelsolin can desensitize CD3-activated T lymphocytes and induce the apoptosis of CD8^+^ T cells in PCa nests

Pathologically, CD3-positive lymphocytes in PCa nests were positive for gelsolin. However, lymphocytes rarely express gelsolin (Supplementary Figures 2 and 3). Moreover, lymphocytes that had not yet entered tumor nests (as shown in Figure 1F), were gelsolin-negative, indicating that lymphocytes might gain gelsolin during their infiltration of PCa nest. In Western blotting analysis of peripheral white blood cells from healthy donors, although a weak signal was detected in white blood cell fractions, gelsolin was not identified in either CD4^+^ or CD8^+^ T cells selected by CD3 antibody-conjugated affinity columns (Supplementary Figure 3).

**Figure 4:**
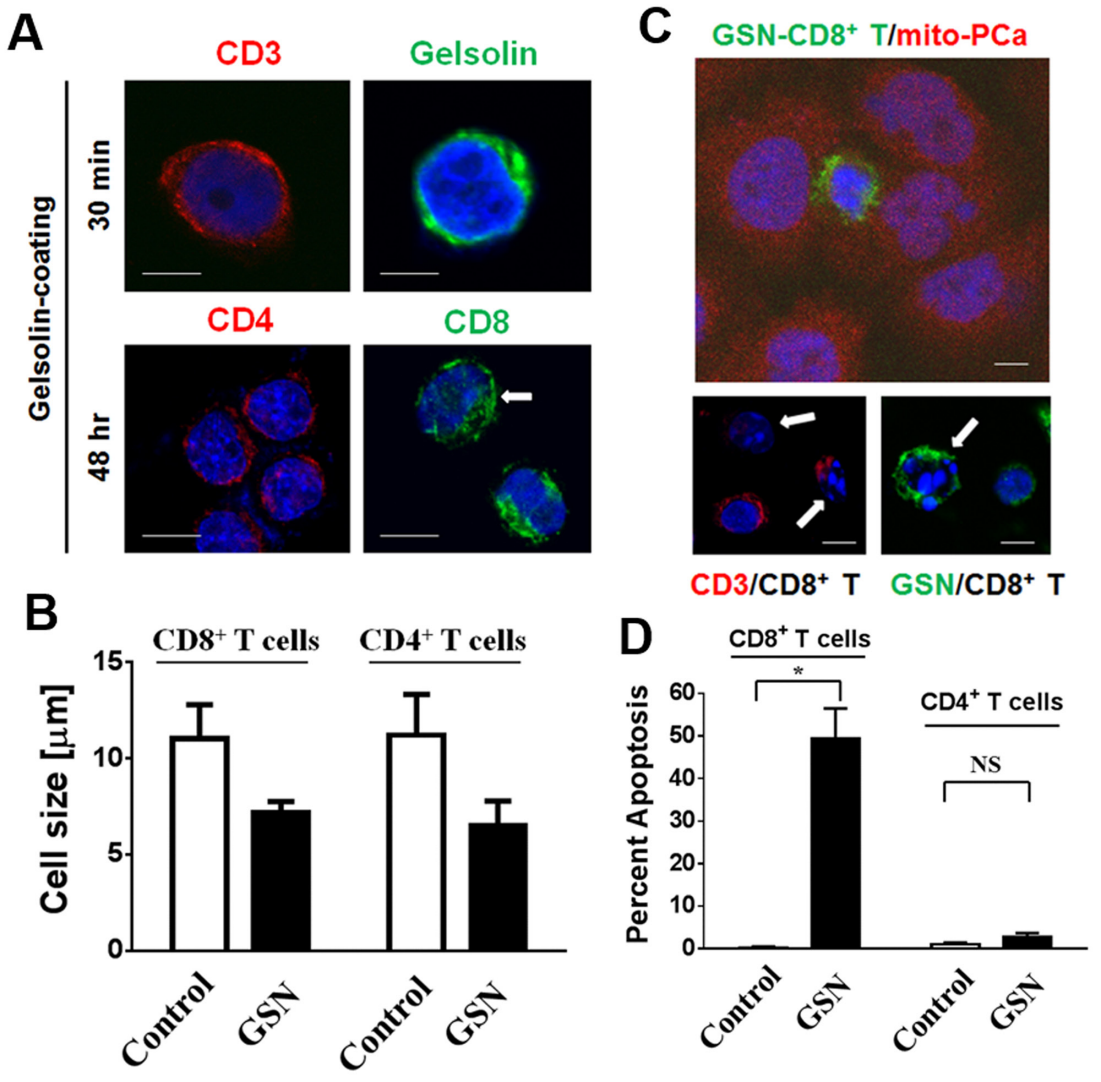
Secreted gelsolin desensitizes CD3-activated CD4^+^ T cells and induces the apoptosis of CD8^+^ T cells in the presence of PCa cells **(A)** Following the incubation of CD3 antibody-activated CD4^+^ and CD8^+^ T cells with conditioned media from LNCaP cells for 30 minutes, both cell types were positive for gelsolin, indicating that gelsolin could indeed bind to the surfaces of T cells (upper panel). **(B)** After being coated with gelsolin for 48 hours, both cell types shrank from about 11.12 ± 0.08 μm to 6.85 ± 0.35 μm in size (*p* < 0.007). **(C)** Gelsolin-coated lymphocytes were co-incubated with PCa cells, **(D)** Apoptosis of CD8^+^ T cells increased about 50% following gelsolin coating, suggesting that gelsolin might be involved in the inactivation and apoptosis of lymphocytes. The cytotoxic effect of PCa cells on CD4^+^ T cells, however, was not as evident. The white bar indicates 5 μm.

Following incubation of CD3 antibody-activated CD4^+^ and CD8^+^ T cells with conditioned media from LNCaP cells (containing 474.7 ± 30.6 μg/mL of secreted gelsolin), both types of T cells became positive for gelsolin (Figure 4A). Interestingly, after being coated with gelsolin for 48 hours, both types of T cells shrank from 11.12 ± 0.08 μm to 6.85 ± 0.35 μm in size (*p* < 0.007, Figure 4B). When gelsolin-coated lymphocytes were co-incubated with PCa cells, the number of apoptotic CD8^+^ T cells increased about 50% (Figure 4C and 4D). The cytotoxic effect, however, was less evident in CD4^+^ T cells. The respective and the joint effects of secreted gelsolin and cancer cells on CD4^+^ and CD8^+^ T lymphocytes suggested that surface markers were involved in a cell-to-cell interaction.

### Extracellular gelsolin inhibits lymphocyte activity by binding to sortilin, CD37 and Fas

**Figure 5:**
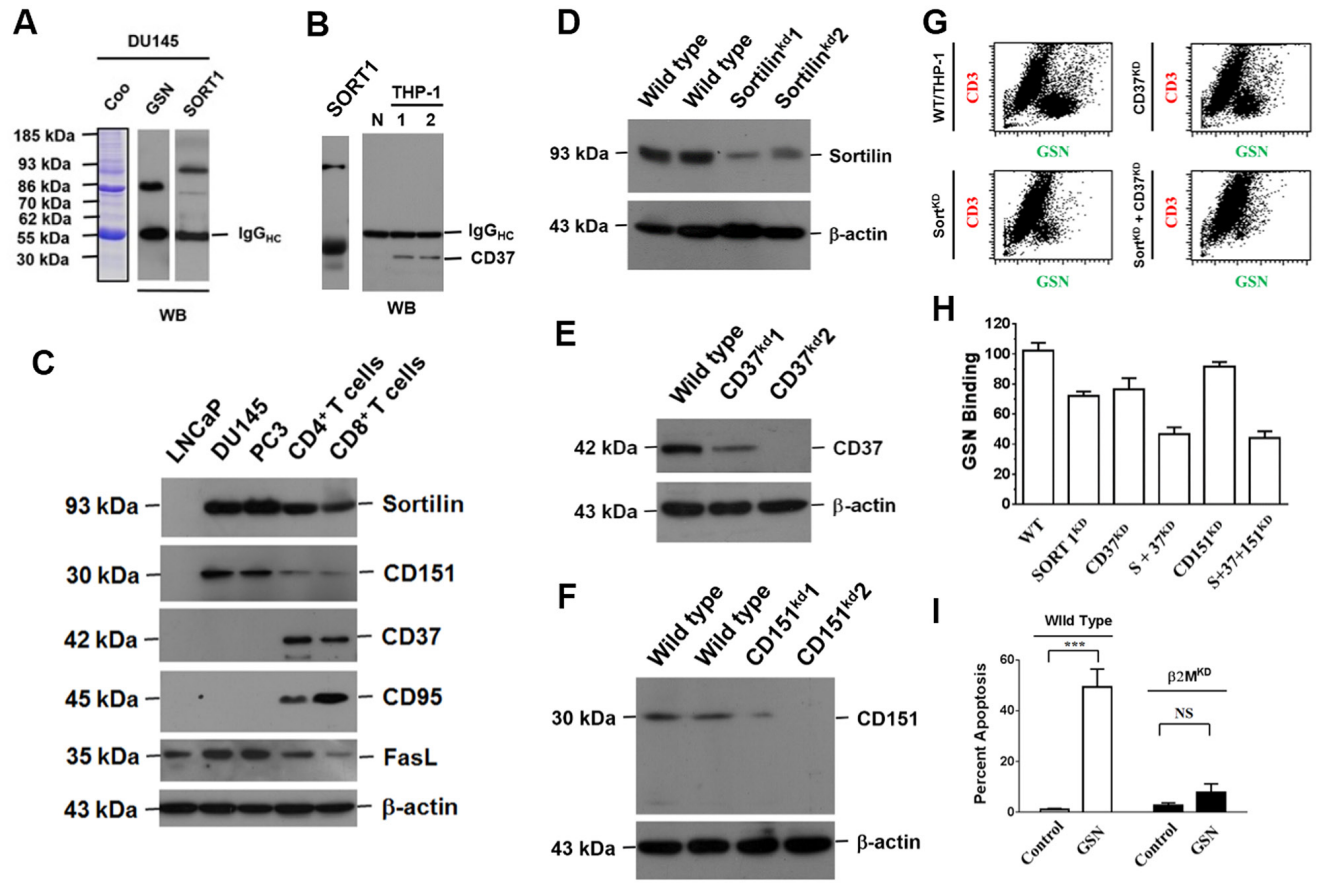
Extracellular gelsolin inhibits lymphocyte activity by binding to sortilin, CD37 and Fas **(A)** Antibodies specific to gelsolin were used to co-precipitate sortilin in the membrane lysates of PCa cells. **(B)** By the same immunoprecipitation method, CD37 was co-precipitated from THP-1 cells. **(C)** The protein expression patterns in PCa cells and lymphocytes, as revealed by Western blotting analysis. Although FasL (CD95L) was detected in all the cells, its receptor (Fas) was predominantly detected in CD8^+^ lymphocytes. Less Fas was detected in CD4^+^ lymphocytes. CD37 and Fas receptor were not clearly detected in all three PCa cell lines. **(D)** Knockdown of sortilin (sortlin^KD^), (**(E**) CD37 (CD37^KD^) or (**(F**) CD151 (CD151^KD^) expression, as revealed by Western blotting. **(G)** Knockdown of CD37 (CD37^KD^) reduced secreted gelsolin (sGSN) binding to CD4^+^ THP-1 cells by 50%. Knockdown of sortilin (Sort^KD^) reduced sGSN binding to CD4^+^THP-1 cells by 75%. When both sortilin and CD37 genes were silenced, the sGSN binding levels reduced to about 10% of the control levels in CD4^+^ THP-1 cells. **(H)** However, knockdown of CD151 (CD151^KD^) only slightly reduced sGSN binding to THP-1 cells. **(I)** Knockdown of β2 microglobulin (a constituent of MHC-I) in PCa cells markedly reduced the apoptosis of CD8^+^ T cells after the two cell types were cultured together.

To identify the membrane markers participating in such a cell-to-cell interaction, we used gelsolin-specific antibodies to precipitate cell surface proteins. Sortilin and CD37 were precipitated from PCa cells and THP-1 cells, respectively (Figure 5A and 5B). Although Fas ligand (FasL, also named as CD95L) was detected in all the cells examined, its receptor, Fas, was predominantly detected in CD8^+^ lymphocytes (Figure 5C). Fas was detected at lower levels in CD4^+^ lymphocytes, and was not detected in cancer cells (Figure 5C) [18]. PCa cells did not express CD37 (Figure 5C). Silencing of sortilin and/or CD37 expression in CD4^+^ THP-1 cells reduced the binding of secreted gelsolin (Figure 5D, 5E, 5G, and 5H). Silencing of CD151, however, did not evidently affect the binding of secreted gelsolin to THP-1 cells (Figure 5F and 5H).

The distinct induction of cell death by secreted gelsolin in CD8^+^ but not CD4^+^ T cells clearly indicated that the presence of Fas and FasL, could not be the sole cause of the lymphocytic death. In fact, in addition to the markers CD4 and CD8, the major difference between CD4^+^ and CD8^+^ T cells is the corresponding molecule to the T cell receptor, that is, major histocompatibility complex II (MHC-II) on antigen-presentation cells, and MHC-I on nucleated cells (in this case, PCa cells) [19]. Silencing of β2-microglobulin (a constitutive of MHC-I) in PCa cells markedly reduced apoptosis of CD8^+^ T cells (Figure 5I). Thus it appeared that the secreted gelsolin-related death of CD8^+^ T cells was cancer cell-specific and could be executed during cell contact.

### Intracellularly, sortilin binds to WASF3 and ATAD3A to promote the intake of cholesterol

**Figure 6:**
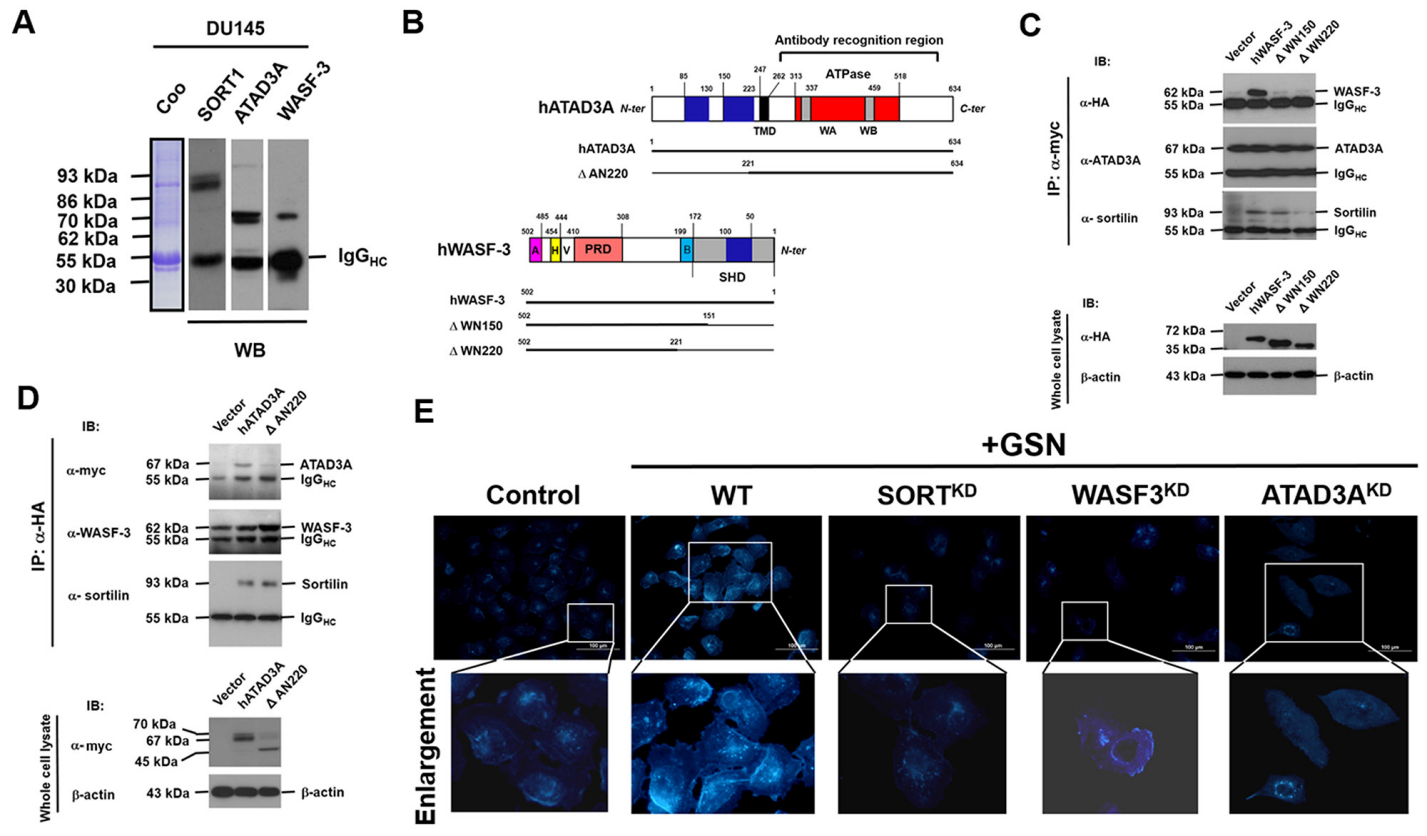
Intracellularly, sortilin binds to WASF3 and ATAD3A to promote the intake of cholesterol **(A)** Antibodies specific to sortilin, were used to co-precipitate ATAD3A and WASF3 from the cytoplasmic proteins of DU145 cells. **(B)** Various deletion mutants were constructed for ATAD3A and WASF3, which were respectively tagged with *c*-*myc* and human influenza hemagglutinin (HA), and the interactions between these proteins were examined. **(C)** Only full-length WASF3 co-precipitated with ATAD3A and sortilin (upper panel). ATAD3A did not co-precipitate with the deletion mutants DWN150 (without N-terminal 150 amino acid residues) and DWN220 of WASF3, although sortilin still co-precipitated with DWN150. The lower panel demonstrates the protein levels of the WASF3 mutants. **(D)** Wild-type ATAD3A co-precipitated with WASF3 and sortilin (upper panel). The deletion mutant DAN220 lost the ability to co-precipitate with WASF3 but not sortilin. The lower panel depicts the protein levels of ATAD3A. **(E)** Filipin III (FR4767, Sigma) fluorescent staining of cholesterol and lipoproteins. In control cells, filipin III fluorescence was detected from the plasma membrane to the nuclear envelope. The addition of secreted gelsolin (sGSN) increased the total amount of filipin III fluorescence, indicating that gelsolin increased the intake of cholesterol and lipoproteins. Knockdown of sortilin (SORT^KD^), WASF-3 or ATAD3A reduced filipin III fluorescence. In WASF3^KD^ cells, though some filipin III staining was evident on the plasma membrane (enlarged portion of WASF3^KD^ cells), the total intensity of filipin III staining was significantly reduced. In ATAD3A^KD^ cells, although some filipin III fluorescence was detected in endosome-like vacuoles, the total fluorescence intensity was also reduced (enlarged portion of ATAD3A^KD^ cells). Interestingly, filipin III fluorescence was not detected in autophagosome-like structures.

In view of the secreted gelsolin could bind to sortilin on PCa cells, we next used antibodies specific to sortilin to react with whole-cell lysates of DU145 cells. The antibodies co-precipitated both ATAD3A and Wiskott-Aldrich syndrome protein family member 3 (WASF-3, also known as WASp family verprolin-homologous protein 3 or WAVE3) from the cytoplasmic proteins (Figure 6A). Next, various deletion mutants of ATAD3A and WASF3 were generated and tagged with C-terminal sequences of *c*-*myc* and human influenza hemagglutinin protein, respectively (Figure 6B). Analysis of these mutants revealed that a basic stretch of WASF3 could bind to sortilin (Figure 6B-6D). On the other hand, WASF3 and ATAD3A could bind to one another through their respective coiled-coil domains (Figures 6B-6D), suggesting that WASF3 might act as an intermediary to bring sortilin and ATAD3A together to facilitate material import. This would be an additional function of ATAD3A, aside from its function in the ER and mitochondria [7, 15].

Amino acid sequence analysis with online software (http://web.expasy.org/protscale/) indicated that sortilin contains several hydrophobic regions, which could bind to lipoproteins and cholesterol (Supplementary Figure 4) [20]. Likewise, secreted gelsolin also contains five lipid binding sites [21], suggesting that both proteins might be involved in the cellular uptake of lipids. Interestingly, the addition of gelsolin increased the total staining intensity of filipin III (Sigma-Aldrich), a fluorescent polyene macrolide antibiotic specific for binding to cholesterol and lipoproteins (Figure 6E). Knockdown of sortilin (SORT^KD^) reduced filipin III staining (Figure 6E). In WASF3^KD^ cells, though some filipin III staining was detected on the plasma membranes (Figure 6E, enlarged portion of WASF3^KD^ cells), the total intensity of filipin III staining was significantly reduced. In ATAD3A^KD^ cells, although some filipin III staining was detected in intracellular vacuoles, the total intensity of filipin III staining was lower than in control cells (Figure 6E, enlarged portion of ATAD3A^KD^ cells).

### The effect of AF38469, a small molecule inhibitor of sortilin, on the expression of gelsolin, sortilin and ATAD3A

**Figure 7:**
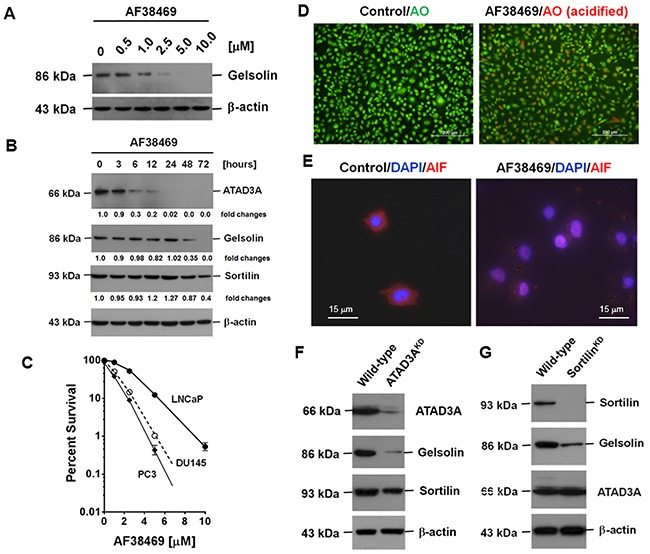
The effect of AF38469, a SMI of sortilin, on the expression of gelsolin, sortilin and ATAD3A **(A)** Gelsolin expression was inhibited in a dose-dependent manner by AF38469. **(B)** AF38469 inhibited gelsolin expression in a time-dependent manner, and also suppressed the expression of ATAD3A and sortilin. The inhibition of ATAD3A expression occurred prior to that of gelsolin and sortilin. **(C)** AF38469 induced PCa cell death, with a median cytotoxicity 2.5 μM for LNCaP, 1.0 μM for DU145 and 0.82 μM for PC3 cells. AF38469 also induced (**(D**) autophagy (as determined by an increase of acridine orange staining) and (**(E**) nuclear translocation of AIF, a molecular signature of apoptosis (as assessed by an increase of nuclear immunocytochemical staining). **(F)** Silencing of ATAD3A clearly reduced gelsolin expression, but only marginally affected sortilin. **(G)** Silencing of sortilin, on the other hand, reduced gelsolin expression, but did not affect that of ATAD3A. Supplementary Materials for “Secreted gelsolin desensitizes and induces apoptosis of the infiltrated lymphocytes in the prostate cancer”

Using gelsolin as a target to screen small molecule inhibitors (SMIs), we identified five potential SMIs: AF38469, an inhibitor of sortilin, suberanilohydroxamic acid (SAHA), and molecules from *A. propinquus* (astragalin), *L. erythrorhizon* (shikonin), and *Folium Mori* (resveratrol) [22]. The latter four compounds have been shown to induce autophagy [23–25]. In this study, we focused on the effects of AF38469, and found that AF38469 inhibited gelsolin expression in a dose- and time-dependent manner (Figure 7A and 7B). AF38469 also inhibited ATAD3A and sortilin expression (Figure 7B); however, the inhibition of ATAD3A expression occurred prior to that of gelsolin and sortilin. AF38469 also induced PCa cell death, with a median cytotoxicity of 2.5 μM for LNCaP, 1.0 μM for DU145 and 0.82 μM for PC3 cells (Figure 7C). This indicated that AF38469 which has a cyclic structure, could be a substrate of AKR1C2 as well [5]. AF38469 also induced autophagy and the nuclear translocation of apoptosis-inducing factor (AIF), another authentic signature of apoptosis (Figure 7D and 7E). Moreover, at a higher concentration of or longer exposure to AF38469, detachment of cells was frequently detected, suggesting that the SMI also repressed the expression of attachment molecules.

Silencing of ATAD3A evidently reduced gelsolin expression, but slightly decreased sortilin expression. On the other hand, silencing of sortilin reduced gelsolin expression, but had no effect on ATAD3A (Figure 7F and 7G), suggesting that the two intracellular transport pathways could converge at a yet-to-be determined site inside the cell (Supplementary Figure 5). At this particular crossing point, the interruption of material transport by SMIs or pathological conditions could initiate autophagy [7, 15].

## DISCUSSION

Two forms of gelsolin protein, a 68-kDa cytosolic form and an 86-kDa secreted form, have been detected [12]. In our Western blotting analyses, the dominant type of gelsolin detected in PCa was an 86-kDa secreted form. Pathology studies further demonstrated that the overexpression of gelsolin in patients with PCa was associated with significantly higher incidence of early tumor recurrence and metastasis, as well as reduced patient survival.

In earlier studies, fluorescent protein-conjugated transfectants were used to investigate the involvement of cytosolic gelsolin in cell movement [11, 13]. We found that the cytosolic gelsolin level was about 1/1000^th^ of the secreted gelsolin level, suggesting that secreted gelsolin could be more significant in PCa. Western blotting analyses, immunofluorescence confocal microscopy, and an enzyme-linked immunosorbent assay revealed that secreted gelsolin is synthesized inside the ER and secreted into extracellular spaces through the intracellular export system, including the Golgi apparatus and transport vesicles (Supplementary Figure 1) [8]. In the normal prostate, secreted gelsolin is secreted into the lumens of the secretory ducts. However, in disease state, especially PCa, some of gelsolin is continuously secreted into extracellular matrices instead of the secretory ducts. Because secretion is limited by the pathological conditions, most of the gelsolin could remain inside cancer cells in the transport vesicles.

Interestingly, amyloid precursor protein (APP), a transmembrane protein, shares some homology to sortilin, a putative receptor for secreted gelsolin, especially in the Aβ40/42 peptide region, where some of the amino acid residues are embedded in the membrane lipids (Supplementary Figure 6). An elegant study by Hirko et al. demonstrated that secreted gelsolin (or, in their terms, plasma gelsolin) reduced the formation of Aβ40/42 peptide from APP [26], suggesting that secreted gelsolin might bind to APP and protect it from γ-secretase cleavage [27]. Our data indicated that, in addition to binding to sortilin, secreted gelsolin could also bind to CD37 (a tetraspanin), which conglomerates with sortilin in lipid rafts on the plasma membrane [28]. By binding to CD37, secreted gelsolin inhibited lymphocyte activity.

It is worth noting that sortilin contains several hydrophobic stretches (Supplementary Figure 4), which may serve as docking sites where low density lipoprotein can unload essential lipids such as dehydroepiandrosterone, ω-3 and, ω-6 fatty acids and cholesterol onto the lipid rafts of target cells [29, 30]. A membrane invagination could then be activated to initiate endocytosis upon the binding of secreted gelsolin to sortilin. Following endocytosis, the essential lipids could be brought to target organelles to sustain cellular functions and supply the lipids required for the aggressive progression of tumor cells. By demonstrating that secreted gelsolin increased the cellular levels of cholesterol and lipoproteins (filipin III-positive fluorescence), our results support such hypothesis. Given our prior findings that ATAD3A is important for the transport of materials such as PSA [8], the present results could be evidence of an intracellular cycle to orchestrate cell proliferation, movement and invasion [15].

The conversion of dehydroepiandrosterone to dihydrotestosterone by dihydrodiol dehydrogenases would become an endogenous source of dihydrotestosterone to perpetually activate androgen receptors in PCa cells [5]. Although we are less certain of where and how the import and the export systems converge, the results of Teng et al. [31] clearly support our findings [5, 7, 8, 15, 32] by demonstrating that ATAD3A collaborates with glucose-regulated protein 78 to modulate the activity of the metastasis-promoting protein WASF-3. A study by You et al. [32], further confirmed that ATAD3A is involved in the intracellular transport of materials, including proteins attached to the ER (e.g., Ataxia telangiectasia mutated protein kinase) and the plasma membrane (e.g., EGFR). Our study model also provides a reasonable explanation for translocation of ER membrane-integrated proteins (such as TREX1, a 3’ DNA repair exonuclease) to the nucleus [33, 34].

WASF-3 is a member of a multiprotein complex, and connects receptors, organelles, and actin *via* its verprolin homologous domain, a coiled-coil at N-terminus and a unique basic stretch (^175^KR^K^/_R_QK^180^) in the middle of the protein. When the verprolin-homologous domain of WASF-3 starts to approach the nucleation core Arp2/3 complex for actin polymerization [33], it is possible that the coiled-coil interacts with the coiled-coils of ATAD3A [7], while the basic segments binds to the acidic C-terminus of sortilin to direct endosomal formation (Supplementary Figure 7), and to provide intracellular anchorage for the oriented movement of cells [29, 35, 36].

In fact, an elegant study by Zhang et al. demonstrated that injection of gelsolin could alleviate severe burn-related brain inflammation, and suggested that systemic gelsolin could desensitize lymphocytes by a yet-to-be determined function [37]. Our data provide not only insights into their findings, but also a mechanism whereby secreted gelsolin desensitizes cells, by binding to sortilin and tetraspanins. Nevertheless, for the induction of apoptosis in CD8^+^ T cells, both FasL and MHC-I on PCa cells were required in addition to sortilin, which is essential for forming lipid rafts to consolidate interactions of receptors.

Using these potential tumor markers to screen SMIs [23], we successfully identified several candidates, including AF38469 (vs. sortilin) and shikonin (vs. ATAD3A). These compounds block the intracellular circulation of essential materials, and curtail imperative supplies required for the proliferation, mobility, and tissue invasion of cancer cells (Supplementary Figure 8). SMIs could inhibit the release of secreted gelsolin, prevent the activation of sortilin-related endocytosis and impede the transport of essential lipids to damage membrane integrity [5, 7, 8, 15, 23, 32]. For these reasons, our results strongly support the possibility of using gelsolin as a target of therapy for PCa.

## MATERIALS AND METHODS

### Cell lines

Three PCa cell lines (PC3, DU145 and LNCaP), which were obtained from American Type Culture Collection (ATCC, Manassas, VA, USA) were used for study. Cells were grown at 37°C in a monolayer in RPMI 1640 supplemented with 10% fetal calf serum, 100 IU/mL penicillin, and 100 μg/mL streptomycin.

### Immunohistochemical staining and immunoblotting

Paraffin sections were stained by an immunoperoxidase method [8]. Following incubation with monoclonal antibodies specific to target protein, slides were treated with biotin-labeled goat anti-mouse immunoglobulin and peroxidase-conjugated streptavidin and developed in 3-amino-9-ethylcarbazole. Crimson precipitate was identified as positive staining. For each immunostaining run, non-tumor part of the prostate tissue served as the negative control. Antibodies to gelsolin (#12953) and CD3 (#4443) were purchased from Cell Signaling Technology. Anti-SORT1 (HPA006889) antibody was from SIGMA, and anti-CD37 (ab81601) antibody was from Abcam. Antibodies to sortilin (PA1-18312) and WASF-3 (PA5-30409) were from ThermoFisher Scientific.

The immunoblotting assay was performed with the same antibodies as in the immunohistochemical staining. Briefly, proteins were separated in a polyacrylamide gel. After electrophoresis, proteins were transferred to a nitrocellulose membrane, and the membrane was probed with target protein-specific antibodies. The signal was amplified by biotin-labeled goat anti-mouse IgG and peroxidase-conjugated streptavidin. Target proteins were visualized through the exposure of the membrane to an X-Omat film (Eastman Kodak, Rochester, NY, USA) with enhanced chemiluminescent reagent (NEN, Boston, MA, USA).

### Immunoprecipitation, gel electrophoresis and protein analysis by MALDI-TOF

For the preparation of total cell lysate, 5 × 10^7^ cells/100 μL phosphate-buffered saline (PBS) were mixed with an equal volume of 2 × NP-40 lysis buffer (40 mM Tris-HCl, pH 7.6; 2 mM ethylenediaminetetraacetic acid; 300 mM NaCl; 2% NP-40; and 2 mM phenylmethylsulfonylfluoride). Protein G sepharose^TM^ (Amersham Biosciences AB, Uppsala, Sweden) was pre-washed before being mixed with 500 μg of total cell lysate. The reaction mixture was incubated at 4°C for one hour, and then centrifuged at 800 × g for 1 minute. The supernatant was reacted with 5 μg of the purified monoclonal antibodies and 20 μL of fresh protein G sepharose at 4°C for 18 hours. The reaction mixture was centrifuged at 800 × g for 1 minute, and the supernatant was removed. Then, the precipitate was washed with 1x PBS and dissolved in loading buffer (50 mM Tris, pH 6.8; 150 mM NaCl; 1 mM disodium ethylenediaminetetraacetic acid; 1 mM phenylmethylsulfonylfluoride; 10% glycerol; 5% β-mercaptoethanol; 0.01% bromophenol blue; and 1% sodium dodecyl sulfate). Electrophoresis was carried out on two 10% polyacrylamide gels with 4.5% stacking gels.

One gel was processed for immunoblotting, and the other gel was stained with Coomassie blue [5–8]. Protein bands on Coomassie-stained gel, which corresponding to the immunoblotting-positive bands, were extracted from the gel for identification by matrix-assisted laser desorption/ionization time-of-flight (MALDI-TOF) analysis on a Voyager-DE^TM^ Pro Biospectrometry Workstation (Applied Biosystems, Milpitas, CA, USA). Fragments of peptide fingerprints were matched with those on the SwissProt database by MS-fit (ProteinProspector 4.0.5., The Regents of the University of California). After electrophoresis, proteins on the first gel were transferred to a nitrocellulose membrane for immunoblotting. The membrane was probed with specific antibodies. The signal was amplified by biotin-labeled goat anti-mouse IgG, and peroxidase-conjugated streptavidin. The protein was visualized through the exposure of the membrane to an X-Omat film (Eastman Kodak, Rochester, NY) with enhanced chemiluminescent reagent (NEN, Boston, MA).

### Inhibition of gene expression by lentivirus transfection of siRNA

Specific gene expression was inhibited using a siRNA method [5, 7, 8, 32]. The siRNA-containing lentiviruses were purchased from Academia Sinica, Taipei, Taiwan.

### Cytotoxicity assay

Cells were seeded at 1000, 2500, and 5000 cells/well, 18 hours prior to drug challenge. The cells were then treated continuously with various drug concentrations for 72 hours. The control group was only treated with the drug diluent, PBS or dimethyl sulfoxide. Culture media were replaced with PBS before the addition of 10 μL of WST-1 (BioVision, Mountain View, CA, USA) and incubation was continued for 4 hours. The percentage of surviving cells in the experiment group was quantified and expressed relative to the control group. All procedures were performed in triplicate [8].

### Confocal immunofluorescence microscopy and fluorescence microscopy

For live mitochondrial staining, the cells were incubated with 200 nM MitoTracker^®^ Red CMXRos dye (Molecular Probes, Eugene, OR, USA) at 37°C for 15 min before fixation. Confocal immunofluorescence microscopy was performed as previously described [8, 24]. Briefly, cells on the slides were fixed with 4% paraformaldehyde for 15 min at room temperature and then permeabilise using 0.1% Triton X-100 (GE Healthcare Life Sciences, Pittsburgh, PA, USA) prior to staining with the primary antibodies. After the primary antibodies were washed off, the slides were incubated with Alexa flour 488 or 546-conjugated secondary antibodies. The nuclei were stained using Hoechst 33342 or 4’,6-diamidino-2-phenylindole (DAPI). A cover slip (Marienfeld Laboratory Glassware, Lauda-Königshofen, Germany) was mounted on the cells by using Prolong Gold Antifade Reagent. The slides were examined under a laser scanning confocal microscope (Olympus, FV1000, confocal laser scanning biological microscope, Tokyo, Japan). Images of the cells were analyzed by using FV10-ASW 3.1 software and Adobe Photoshop CS6 (Adobe Systems Incorporated).

For staining cholesterol and lipoproteins, the cells were fixed with 4% paraformaldehyde for 15 min at room temperature. After rinsing with water 3 times, 10 mM glycine was added to quench off paraformaldehyde. The cells were then stained with filipin III (76 μM in PBS with 10% FBS) for 2 hours at room temperature. After rinsing with water 3 times, the slides were examined under a fluorescence microscope (Olympus BX51, Tokyo, Japan). UV fluorescence was activated using X-Cite^®^ 120 Fluorescence Microscope Illumination System (EXFO, Quebec, Canada).

### Preparation of peripheral T lymphocytes

Peripheral blood mononuclear cells were isolated from healthy donors by a Histopaque (Sigma, St Louis, Mo, USA). T lymphocytes were purified by anti-CD4/8-conjugated magnet dynabeads (Dynabeads^®^ CD4/8, Invitrogen Taiwan, Ltd.) [15]. Viable T cells were harvested from the beads by using DETACHaBEAD^®^ CDT (Invitrogen Taiwan, Ltd.). Briefly, peripheral blood mononuclear cells were suspended in PBS with 0.1% bovine serum albumin and then incubated with anti-CD4/8-conjugated magnet dynabeads at 4°C for 20 minutes. The reaction mixture was then placed on a magnet to attract cells expressing CD4/8 on their membrane. Magnet-bound cells were washed four times with PBS and then detached from the beads by DETACHaBEAD^®^ CD4/8. Purity of the isolated cells was determined by immunocytochemistry with anti-CD4 or CD8.

### Flow cytometric analysis

Cells (1×10^6^ cells/well) were collected by centrifugation (800 × g, 5 min). After the supernatant was decanted, the cells were resuspended in PBS with 1% bovine serum albumin, and incubated with fluorescence-labeled antibodies (anti-GSN [#12953, Cell Signaling Technology, Inc.] with phytoerythrin [PE] Cy5-conjugated anti-rabbit, and PE-conjugated anti-CD3 [BD PharMingen, San Diego, CA, USA]) on ice for 30 minutes. Cells were washed twice with PBS containing 0.1% bovine serum albumin before brief fixation with 1% paraformaldehyde on ice. The number of antibody-labeled cells was then determined by flow cytometry (Beckman Coulter Cytomics^TM^ FC500), and data were analyzed using machine-embedded CXP software [15].

### Patients, tissue specimens and pathological slide evaluation

Some of the patients in this study were from a cohort used in a previous study [5, 8]. Briefly, from January 1996 to December 2006, 97 pathologic samples were collected from patients with clinically diagnosed PCa. The pathologic specimens were collected before hormonal or radiation therapy. The Medical Ethics Committee of the China Medical University Hospital approved the protocol, and written informed consent to donate specimens was obtained from each patient. Gleason score, tumor stage, and tumor grade were determined in accordance with American Joint Committee on Cancer guidelines [38]. All patients had undergone radical prostatectomy with bilateral pelvic lymph node dissection. The diagnosis of PCa was confirmed pathohistologically. Patients with lymph node involvement or the presence of tumor-positive margins underwent radiation therapy with 72-78 Greys directed at the afflicted areas. After treatment, all patients were routinely followed up every 3 to 6 months on an outpatient basis. Tumor recurrence and metastasis were diagnosed based on elevated PSA levels (biochemical recurrence) and evidence of disease in whole body bone scans or computerized tomography scans. A tissue microarray of tumor samples from 183American patients with PCa (Ambion Inc., Austin, TX, USA) was used to compare gelsolin expression between Chinese and American patients.

Non-tumorous prostate tissue served as the internal negative control. Slides were evaluated by three independent pathologists blinded to the clinicopathologic variables. An immunoreactive scoring system was adapted for this study [39]. Briefly, a specimen was considered strong positive if more than 25% of cancer cells were positively stained; weak positive if less than 25% were positively stained [7, 8, 23, 24, 32].

### Statistical analysis

Associations between gelsolin expression and clinicopathologic variables were analyzed by the Chi-Square test. The Chi-Square test for trend was used when corresponding variables exceeded two categories. Statistical significance was set at *p* < 0.05. All statistical analyses were performed on a personal computer with the statistical package GraphPad Prism6 for Windows (Version X, GraphPad Software, Inc., La Jolla, CA, USA).

## SUPPLEMENTARY MATERIALS FIGURES AND TABLES



## Abbreviations

ADR: adriamycin;
AIF: apoptosis-inducing factor;
AKR1C2: aldo-keto reductase 1C2;
APP: amyloid precursor protein;
AR: androgen receptor;
ATAD3A: ATPase AAA domain containing 3A;
ATM: Ataxia telangiectasia mutated;
β2M: β2-microglobulin;
BHPE: benign hypertrophic prostate epithelia;
cGSN: cytosolic GSN;
DHEA: dehydroepiandrosterone;
DHT: dihydrotestosterone;
DRP1: dynamin-related protein 1;
EGFR: epidermal growth factor receptor;
ELISA: enzyme-linked immunosorbent assay;
ER: endoplasmic reticulum;
FBS: fetal calf serum;
5-FU: 5-fluorouracil;
GRP-78: glucose-regulated protein 78;
GSN: gelsolin;
HA: human influenza hemagglutinin;
HGF: hepatocyte growth factor;
IL-8: interleukin 8;
KLK3: kallikrein-3;
LDL: low density lipoprotein;
LM: light membrane;
MAM: mitochondria-associated membrane;
MHC-II: major histocompatibility complex II;
MTX: methotrexate;
NTPE: non-tumorous prostate epithelium;
NTS3: neurotensin receptor-3 (sortilin);
PCa: Prostate cancer;
PSA: prostate specific antigens;
SAHA: suberanilohydroxamic acid;
sGSN: secreted GSN;
SMIs: small molecule inhibitors;
TIL: tumor-infiltrated lymphocytes;
UV: ultraviolet;
VHD: verprolin homologous domain;
WASF-3: Wiskott-Aldrich syndrome protein family member 3;
WAVE3: WASp family verprolin-homologous protein 3.
