# Supplementary material for: Secreted gelsolin desensitizes and induces apoptosis of infiltrated lymphocytes in prostate cancer
Source: Oncotarget. 2017 Aug 23;8(44):77152–67. doi: 10.18632/oncotarget.20414 (PMC5652770; doi:10.18632/oncotarget.20414)
Supplement: Supplementary file 1 [file oncotarget-08-77152-s001.pdf]

Secreted gelsolin desensitizes and induces apoptosis of infiltrated lymphocytes in prostate cancer

SUPPLEMENTARY MATERIALS

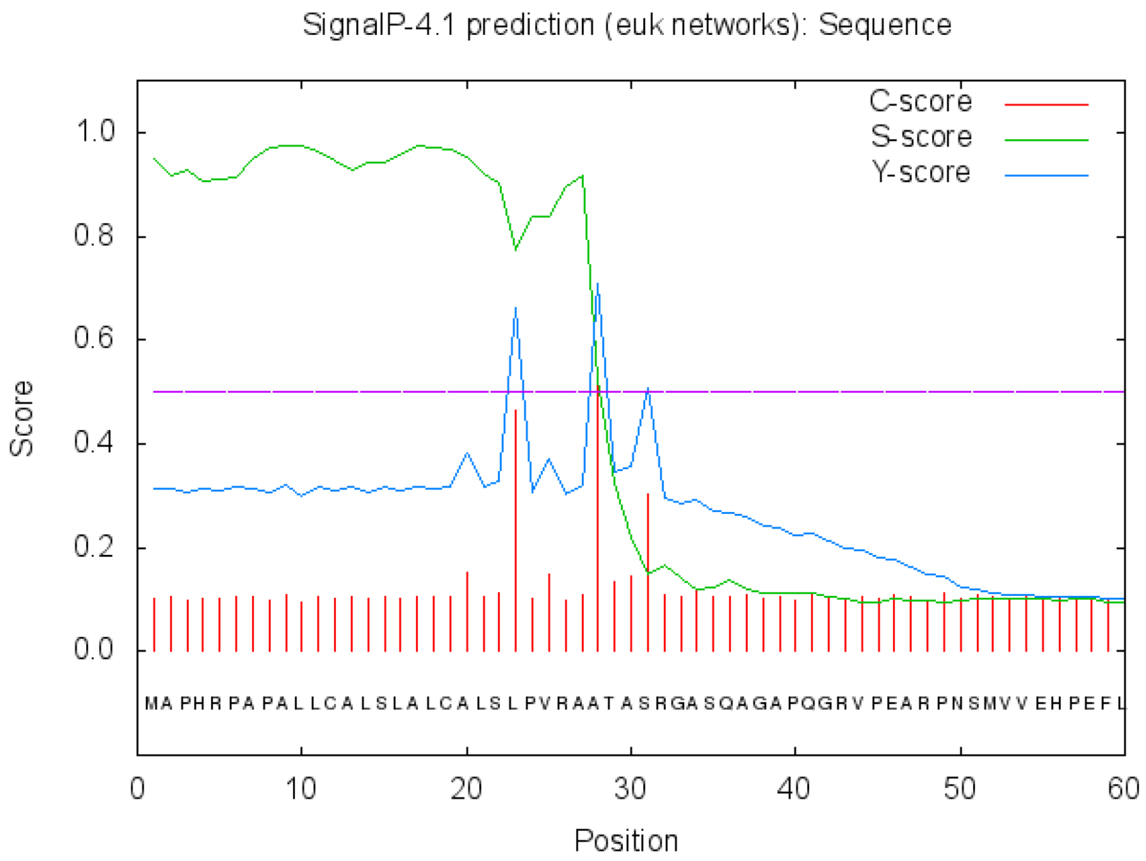

Supplementary Figure 1: Analysis of gelsolin protein by web programs, signal peptide analysis and PSORT II. **A.** Prediction of the presence of signal peptide in gelsolin (http://www.cbs.dtu.dk/services/SignalP/)

# Measure	Position	Value	Cutoff	signal peptide?
D	1-27	0.827	0.450	YES

(B) Results of PSORT II prediction of full length gelsolin.

As predicted by a web program, PSORT II (http://psort.hgc.jp/), the full length gelsolin (fGSN, GenBank: AK315494) is synthesized on the rough endoplasmic reticulum (RER).
The protein is secreted into the extracellular space as secreted gelsolin (sGSN).

<u>GvH: von Heijne's method for signal seq. recognition</u>
GvH score (threshold: -2.1): 3.30
possible cleavage site: between 22 and 23

>>> Seems to have a cleavable signal peptide (1 to 22)

Gavel: prediction of cleavage sites for mitochondrial preseq
R-2 motif at 53 GRV|PE HYPERLINK

ER Membrane Retention Signals:

XXRR-like motif in the N-terminus: APHR

Results of the k-NN Prediction

k = 9/23

77.8 %: extracellular, including cell wall

11.1 %: vacuolar

11.1 %: Golgi

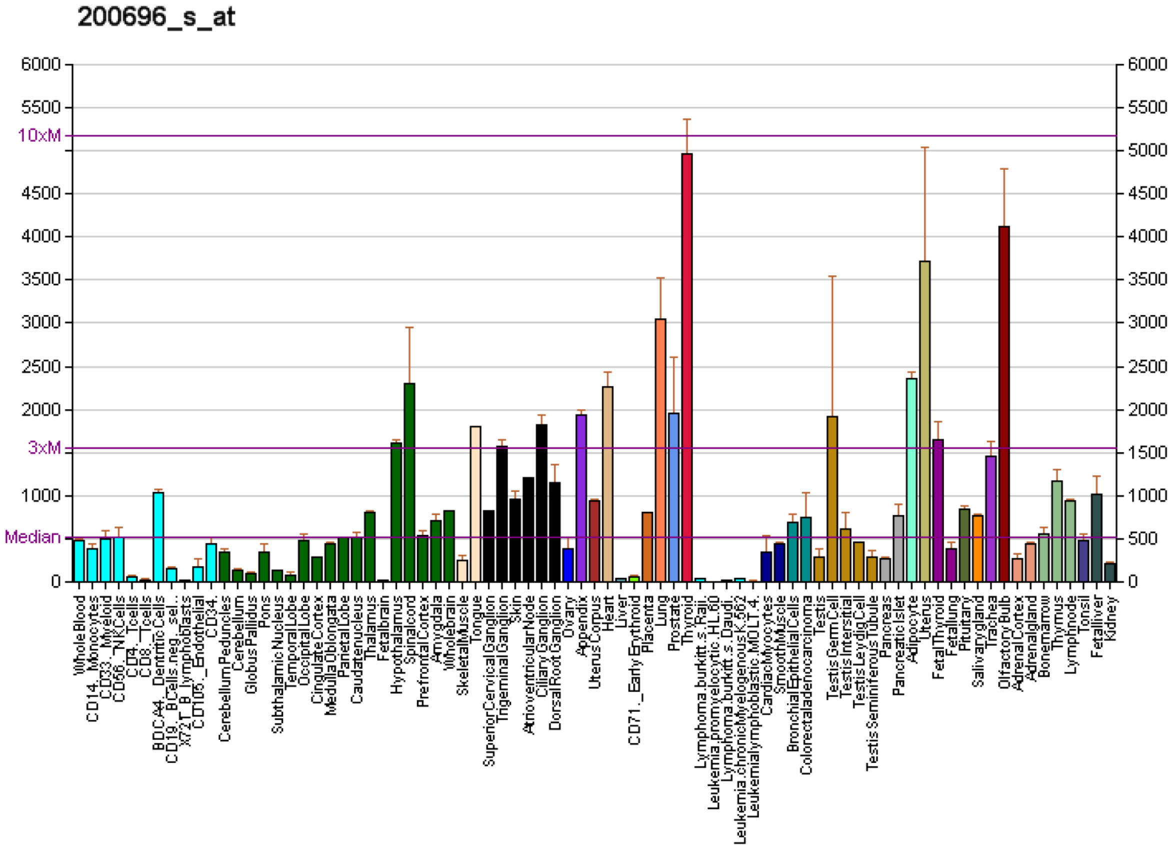

Supplementary Figure 2: Expression of gelsolin protein in human cells, tissues and organs as detected by immunostaining. Besides nerve and endocrine-related cells, both CD4 and CD8 T lymphocytes (columns 5 and 6) as well as myelocytic and lymphocytic leukemia cells rarely express gelsolin protein (https://en.wikipedia.org/wiki/Gelsolin, PBB_GE_GSN_200696_s_at_fs). The following diagram was created by AndrewGNF, based on data from Su AI, Wiltshire T, Batalov S, Lapp H, Ching KA, Block D, Zhang J, Soden R, Hayakawa M, Kreiman G, Cooke MP, Walker JR, and Hogenesch JB. A gene atlas of the mouse and human protein-encoding transcriptomes. Proceedings of National Academy of Science USA. 2004; 101(16): 6062–6067. doi:10.1073/pnas.0400782101. PMID 15075390. Creation supported by the Genomics Institute of the Novartis Research Foundation.

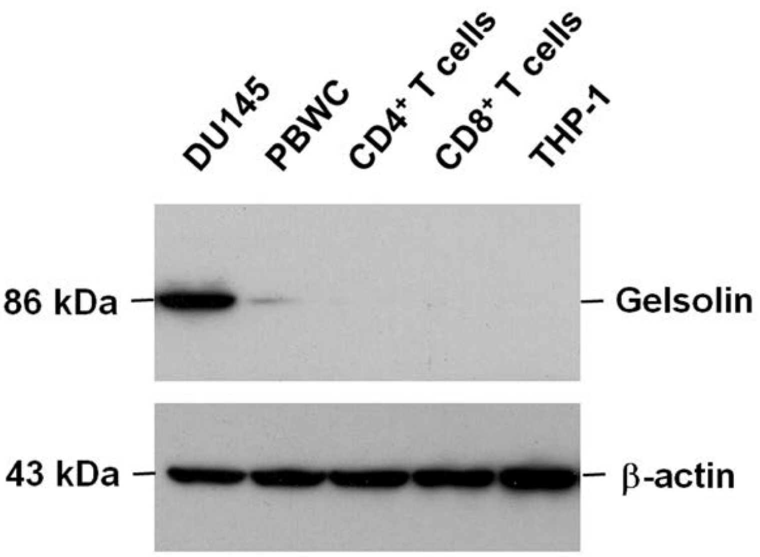

Supplementary Figure 3: Expression of gelsolin protein in human prostate cancer cells (DU145), peripheral white blood cells (PBWC), CD4$^+$ T cells, CD8$^+$ T cells and CD4$^+$ THP-1 cells as detected by Western blotting analysis. Using Western blotting analysis, although a weak signal was detected in the PBWC fraction, gelsolin was not identified in either CD4- or CD8-antibody selected cells. Neither was gelsolin detected in THP-1 (CD4$^+$ T) cells.

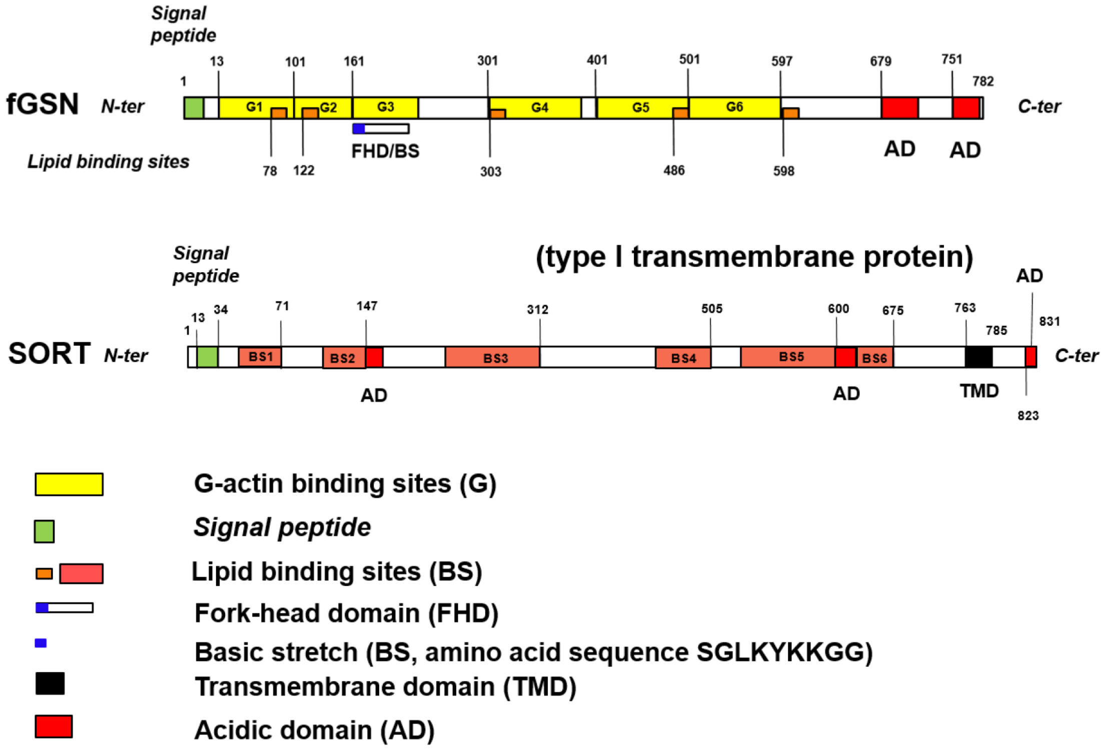

Supplementary Figure 4: Composite sketches of amino acid sequence analysis results of full length gelsolin (fGSN) and sortilin (SORT) by a web software ProtScale (http://web.expasy.org/protscale/). Sortilin contains six hydrophobic domains, which may potentially interact with lipoproteins, lipids and cholesterol. Likewise, sGSN also contained five putative lipid binding sites (Méré J, Chahinian A, Maciver SK, Fattoum A, Bettache N, Benyamin Y, and Roustan C. Gelsolin binds to polyphosphoinositide-free lipid vesicles and simultaneously to actin microfilaments. Biochemistry Journal. 2005; 386(1):47-56.), implicating that both proteins could be involved in cellular uptake of lipids.

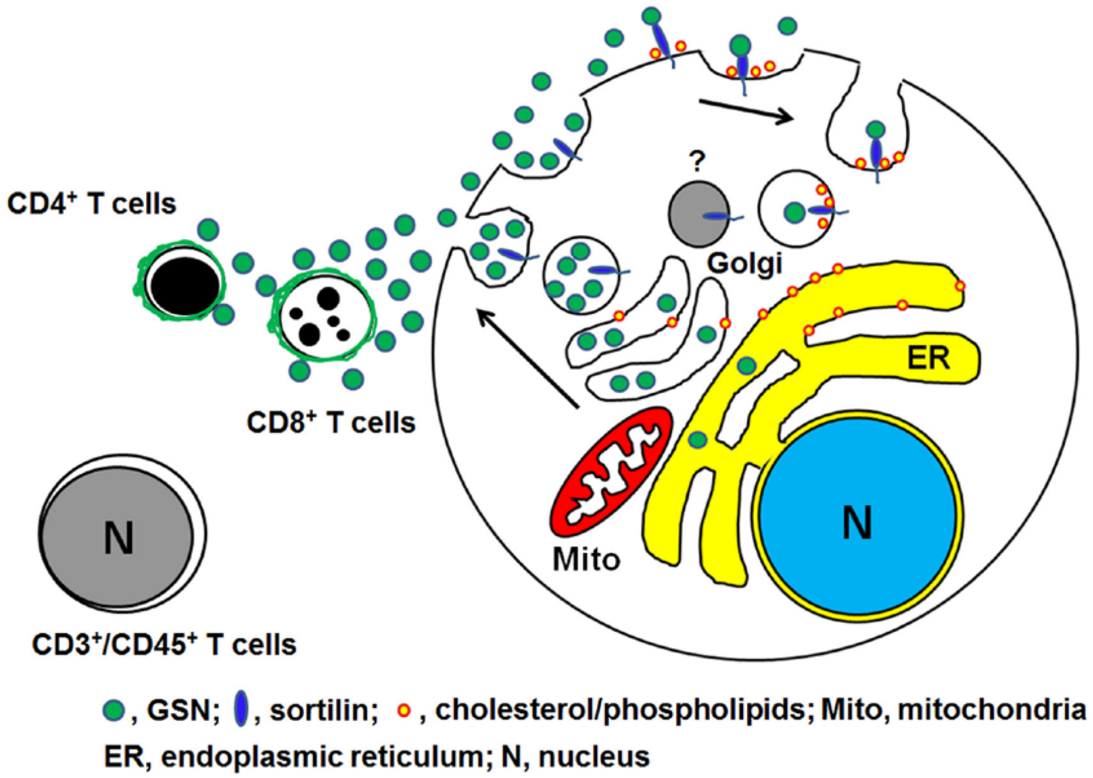

Supplementary Figure 5: The simple sketch of gelsolin secretion, encapsulation and inactivation of lymphocytes, binding to sortilin, as well as induction of endocytosis and apoptosis. Inactivation of lymphocyte is shown by a black nucleus of $CD4^+$ T cell, and apoptosis of lymphocyte is shown by a $CD8^+$ T cell with micro-nucleation. Activated T cell is shown by a $CD3^+/CD45^+$ T cell with round gray nucleus.

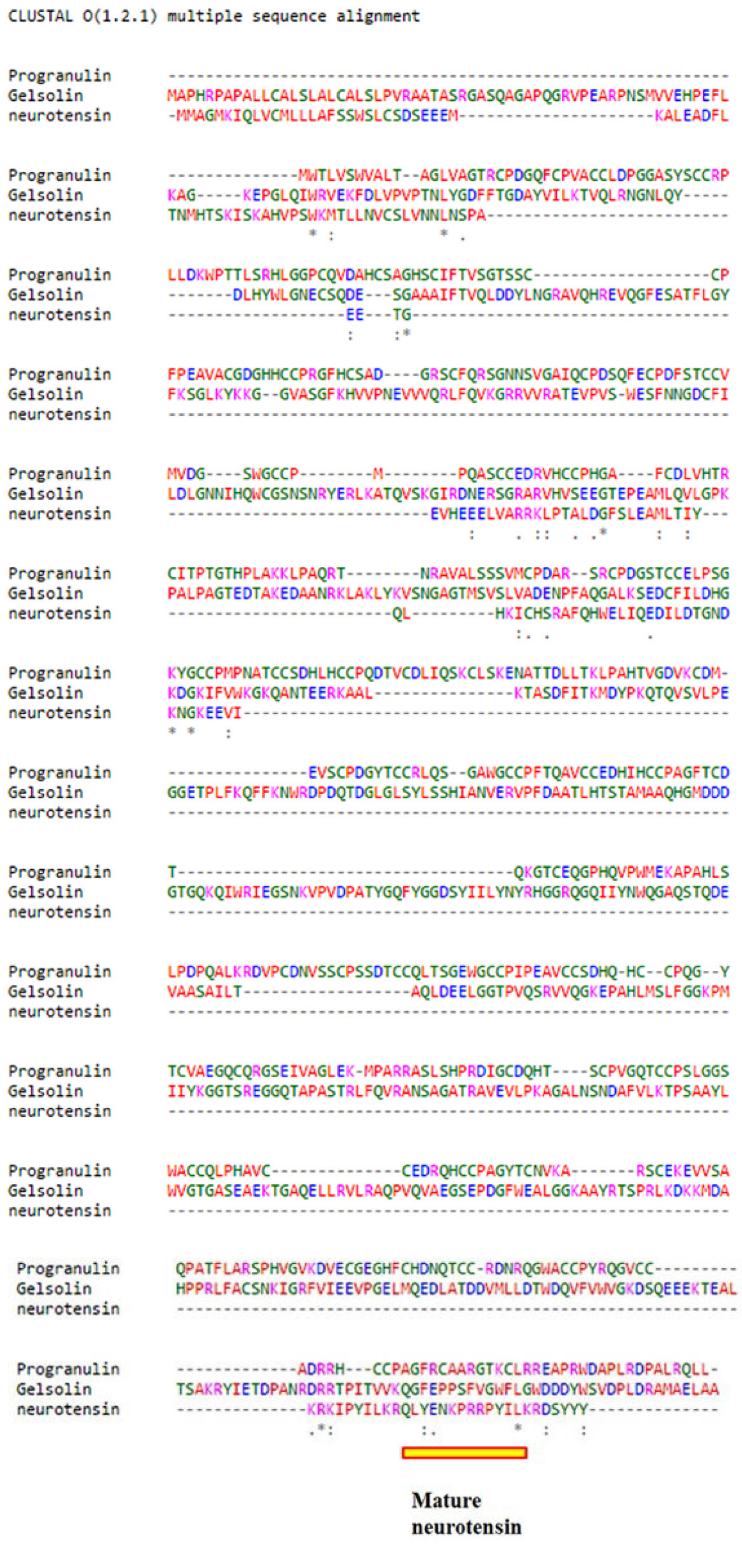

Supplementary Figure 6: Comparison of amino acid sequences among progranulin, gelsolin, and neurotensin. The overlapped region is labeled underneath with yellow stripe. The overlapped amino acid sequence in gelsolin is from a.a. 748 to 561; and progranulin is from a.a. 561 to 573. On both sides of neurotensin homologs, there are two putative coiled-coils cliffs, which may facilitate protein binding to sortilin. (Comparison was done by Clustal Omega, http://www.ebi.ac.uk/Tools/msa/clustalo).

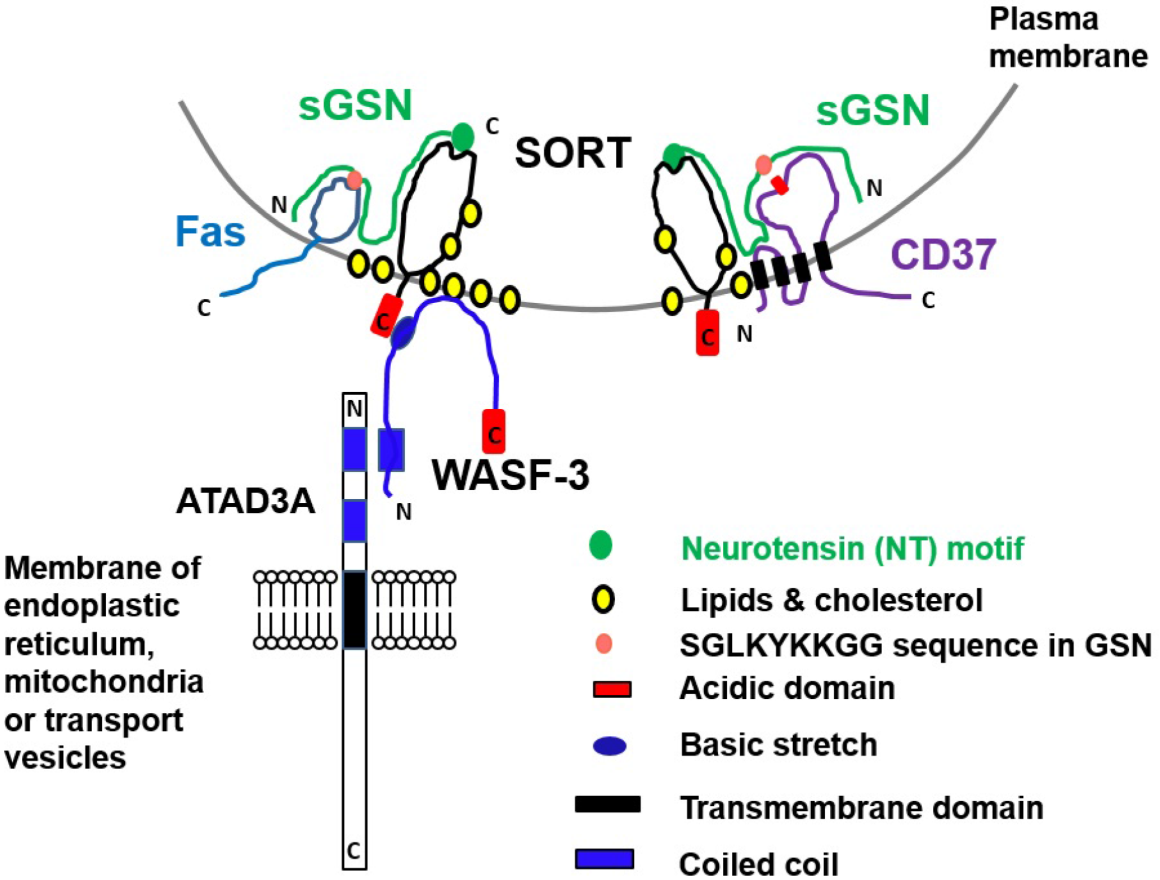

Supplementary Figure 7: The simple sketch of gelsolin binding to sortilin during sortilin activation. WASP-3 connects the activated sortilin and organelles through interactions between basic stretch on the protein and intracellular acidic domain of sortilin as well coiled-coils on both WASF-3 and ATAD3A, which would later bring WASP-3 to mitochondria [Teng Y, Ren X, Li H, Shull A, Kim J, and Cowell JK. Mitochondrial ATAD3A combines with GRP78 to regulate the WASF3 metastasis-promoting protein. Oncogene. 2016; 35(3):333-343].

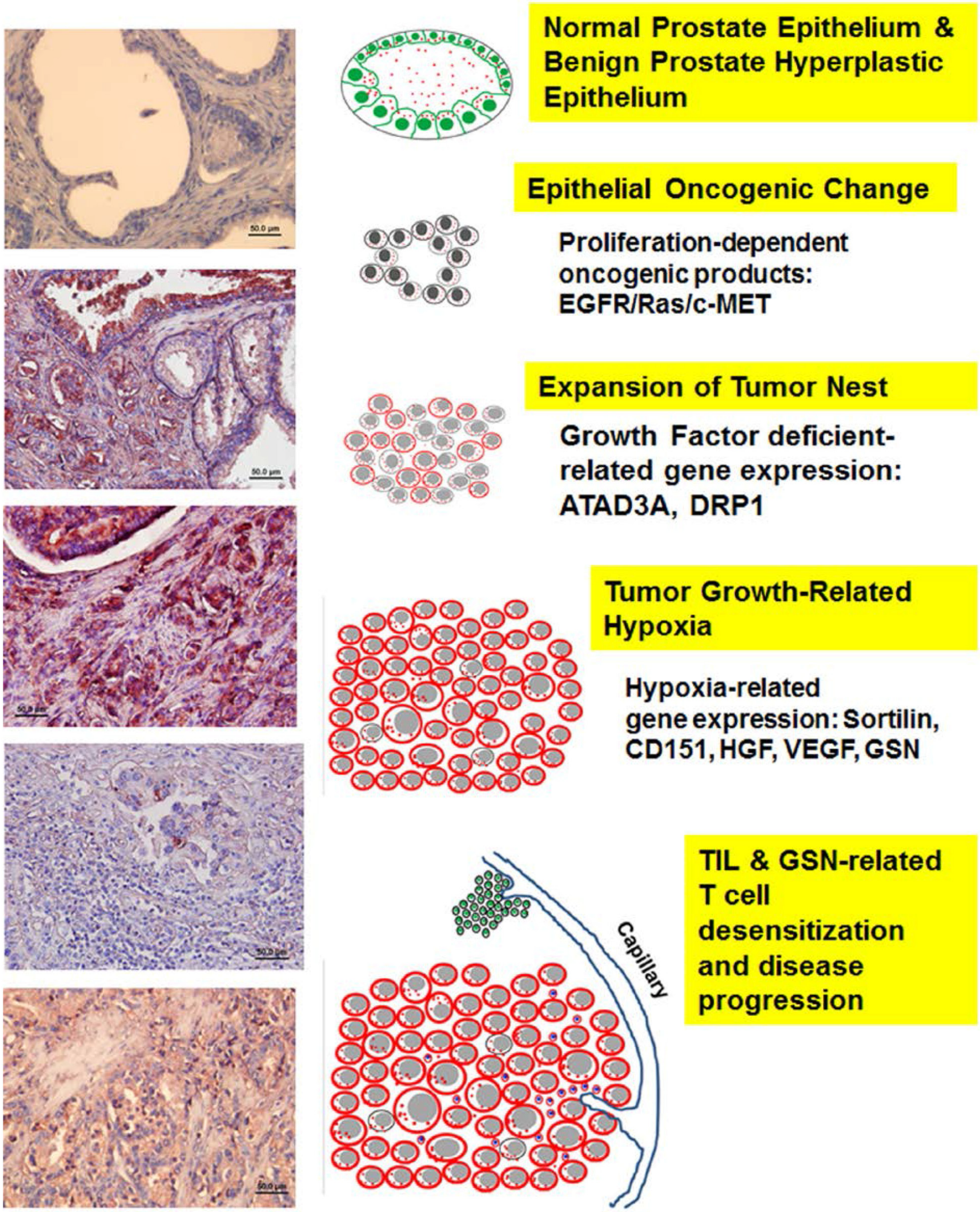

Supplementary Figure 8: The simple sketch of gelsolin expression during different phases of prostate carcinogenesis and progression based on our study results. Green: normal or benign hypertrophic changes; Red: gelsolin; Grey: carcinogenic transformation; Blue: apoptotic T cells.
